# miR675 Accelerates Malignant Transformation of Mesenchymal Stem Cells by Blocking DNA Mismatch Repair

**DOI:** 10.1016/j.omtn.2018.11.010

**Published:** 2018-11-24

**Authors:** Yanan Lu, Shuting Song, Xiaoxue Jiang, Qiuyu Meng, Chen Wang, Xiaonan Li, Yuxin Yang, Xiaoru Xin, Qidi Zheng, Liyan Wang, Hu Pu, Xin Gui, Tianming Li, Dongdong Lu

**Affiliations:** 1 Research Center for Translational Medicine at Shanghai East Hospital, School of Life Science and Technology, Tongji University, Shanghai 200092, China

**Keywords:** miR675, human mesenchymal stem cell, malignant transformation, P62

## Abstract

miR675 is highly expressed in several human tumor tissues and positively regulates cell progression. Herein, we demonstrate that miR675 promotes malignant transformation of human mesenchymal stem cells. Mechanistically, we reveal that miR675 enhances the expression of the polyubiquitin-binding protein p62. Intriguingly, P62 competes with SETD2 to bind histone H3 and then significantly reduces SETD2-binding capacity to substrate histone H3, triggering drastically the reduction of three methylation on histone H3 36th lysine (H3K36me3). Thereby, the H3K36me3-hMSH6-SKP2 triplex complex is significantly decreased. Notably, the ternary complex’s occupancy capacity on chromosome is absolutely reduced, preventing it from DNA damage repair. By virtue of the reductive degradation ability of SKP2 for aging histone H3.3 bound to mismatch DNA, the aging histone H3.3 repair is delayed. Therefore, the mismatch DNA escapes from repair, triggering the abnormal expression of several cell cycle-related genes and causing the malignant transformation of mesenchymal stem cells. These observations strongly suggest understanding the novel functions of miR675 will help in the development of novel therapeutic approaches in a broad range of cancer types.

## Introduction

Mesenchymal stromal cells (MSCs) are a kind of stromal cell within the tumor microenvironment.^1^ Studies have revealed MSCs tended to directionally migrate toward tumor cells.^2^ MicroRNAs are potent regulators of gene expression and modulate multiple cellular processes, including tumorigenesis. miR675 is expressed exclusively in the various tissues.^3^, ^4^ Moreover, H19 maintains hematopoietic stem cell-repopulating ability through a miR-675-IGFR-signaling circuit.^5^ miR675 is associated with carcinogenesis. For example, miR-675 modulates human gastric cancer cell proliferation by targeting tumor suppressor Runt Domain Transcription Factor 1 (RUNX1).^6^ Overexpression of miR675 in hepatocellular carcinoma links to a dramatic upregulation of proliferative and growth capacity.^7^ Intriguingly, miR-675 targeted the 3′ UTRs of the histone deacetylase (HDAC) 4–6 transcripts and resulted in the deregulation of HDACs 4–6, and the CTCF-H19-miR-675-HDAC-regulatory pathway plays an important role in the commitment of bone marrow mesenchymal stem cells (BMSCs) into adipocytes.^8^ Moreover, H19-miR-675-transforming growth factor β1 (TGF-β1)-Smad3-HDAC regulates osteogenic differentiation of human MSCs (hMSCs).^9^miR-675 enhances tumorigenesis and metastasis of breast cancer cells by downregulating c-Cbl and Cbl-b.^10^ However, the exact roles of mature miR-675 in hepatocarcinogenesis have not been identified.

P62 is a multifunctional adaptor protein implicated in selective autophagy.^11^ Recent findings link p62 activity to tumorigenesis.^12^ Interrupting the TRB3-p62 interaction produces potent anti-tumor efficacies against tumor growth and metastasis.^13^ Moreover, P62 upregulation is commonly observed in human tumors and contributes directly to tumorigenesis.^14^, ^15^ Strikingly, P62 is necessary for Ras to trigger IkappaB kinase (IKK) through the polyubiquitination of tumor necrosis factor (TNF) receptor-associated factor 6 (TRAF6).^16^

DNA mismatch repair (MMR) is an important DNA repair pathway that plays critical roles in DNA replication fidelity and genome stability.^17^, ^18^ Cancers deficient in DNA MMR frequently show favorable prognosis. HDAC10 expression is associated with poor prognosis in paracarcinoma tissues with a potential involvement in DNA MMR.^19^ One report shows that miR-1290 may become a promising biomarker of deficient MMR (dMMR) in colon cancer.^20^ Moreover, the genomes of cancers deficient in MMR contain exceptionally high numbers of somatic mutations.^21^

In this study, we demonstrate that miR675 promotes the high expression of the polyubiquitin-binding protein p62 and reduces the H3K36me3-hMSH6-SKP2 ternary complex formation. Thereby, the damaged DNA escapes repair, which causes the malignant transformation of MSCs. This suggests that understanding the novel functions of miR675 will help in the development of new therapeutic approaches that may be useful in a broad range of cancer types.

## Results

### miR675 Accelerates Malignant Growth of MSCs

**Figure 1:**
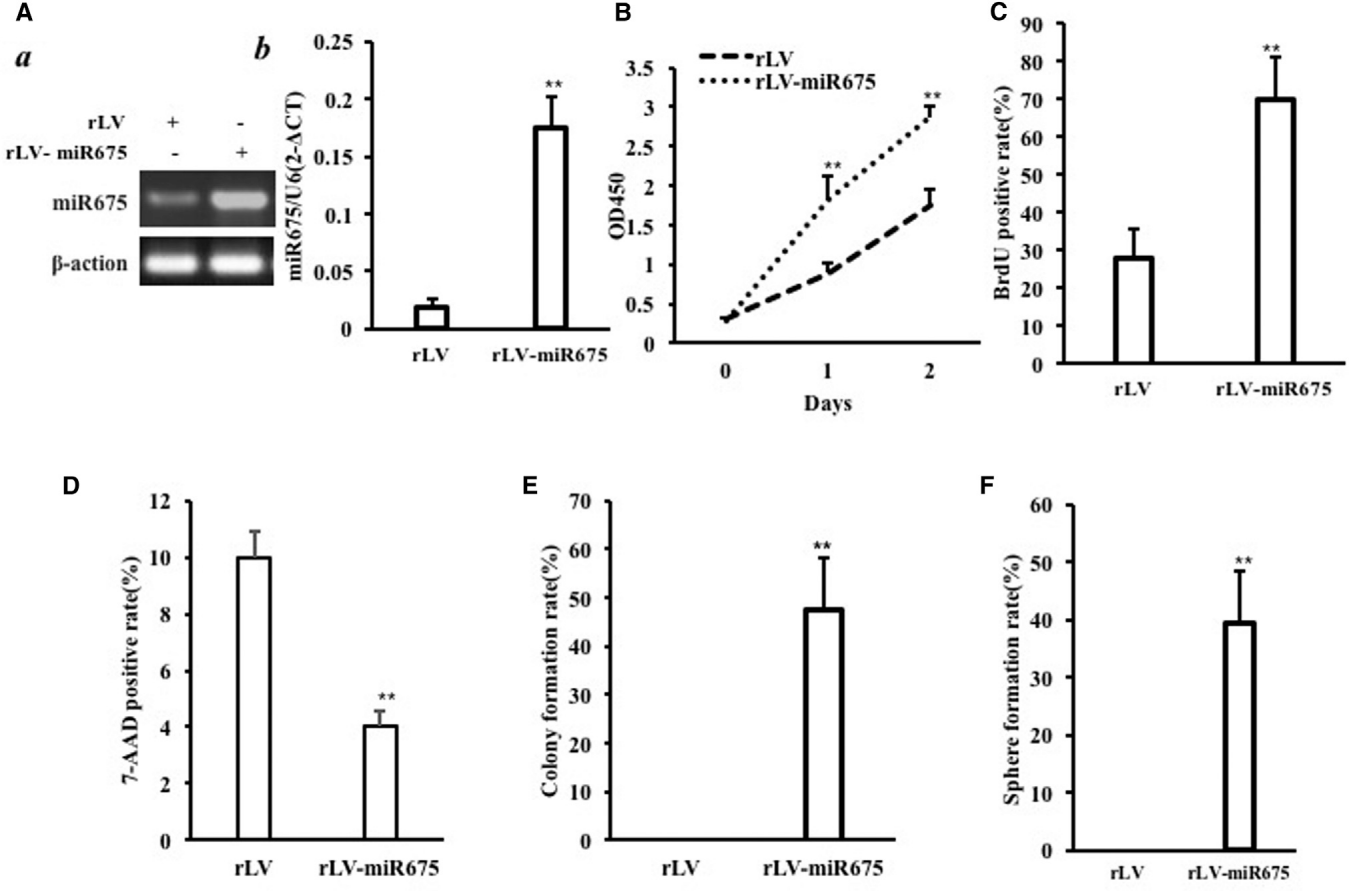
miR675 Accelerates Malignant Growth of Human Mesenchymal Stem Cells *In Vitro* (A) (a) The RT-PCR analysis of pre-miR675 in HBMMSCs infected with rLV and rLV-miR675, respectively. β-actin was the internal control. (b) The real-time RT-PCR analysis of mature miR675 in the HBMMSCs infected with rLV and rLV-miR675, respectively. U6 was the internal control. (B) Cell growth assay using CCK8. Each value was presented as mean ± SEM (n = 3). (C) S phase cell assay using BrdU. Each value was presented as mean ± SEM (n = 3). (D) The assay of flow cytometry using 7-AAD. Each value was presented as mean ± SEM (n = 3). (E) Cell soft agar colony formation assay. Each value was presented as mean ± SEM (n = 3). (F) Cell sphere formation ability. Each value was presented as mean ± SEM (n = 3).

To validate whether miR675 triggers malignant transformation of human bone marrow MSCs (HBMMSCs), we first constructed a stable HBMMSC cell line with overexpression of miR675. The two stable HBMMSC cell lines were established by infecting with rLV and rLV-miR675, respectively. The pre-miR675 and mature miR675 can be expressed in these HBMMSCs (Figures S1 and S2). The successful alteration of miR675 expression in the established HBMMSCs was verified by RT-PCR or real-time RT-PCR. As shown in Figure 1A a, the findings showed that the level of pre-miR675 was increased in the rLV-miR675 group compared to control (p < 0.01). As shown in Figure 1A b, the findings showed that the level of mature miR675 was increased in the rLV-miR675 group compared to control (p < 0.01).

Next, we examined the growth curves of the two HBMMSC cell lines by the CCK8 assay. As shown in Figure 1B, overexpression of miR675 significantly increased the growth ability of HBMMSCs when compared to the control cells. To further address this issue, we detected the S phage cells by bromodeoxyuridine (BrdU) staining. As shown in Figure 1C, excessive miR675 significantly increased the BrdU-positive rate compared to control (69.87% ± 10.95% versus 28.03% ± 7.45%, p = 0.007 < 0.01). The assay of flow cytometry showed that the 7-AAD-positive rate was significantly decreased in the rLV-miR675 group compared to control (9.9967% ± 0.94% versus 4.03% ± 0.53%, p = 0.009474 < 0.01) (Figure 1D). Then we performed soft agar colony formation assay; as shown in Figure 1E, the colony formation efficiency rate was 47.38% ± 10.98% in the rLV-miR675 group, whereas the other group did not exhibit colony formation. Furthermore, our results showed that the self-renewing sphere formation rate was 39.55% ± 9.05% in the rLV-miR675 group, however, the other group did not exhibit sphere formation (Figure 1F).

**Figure 2:**
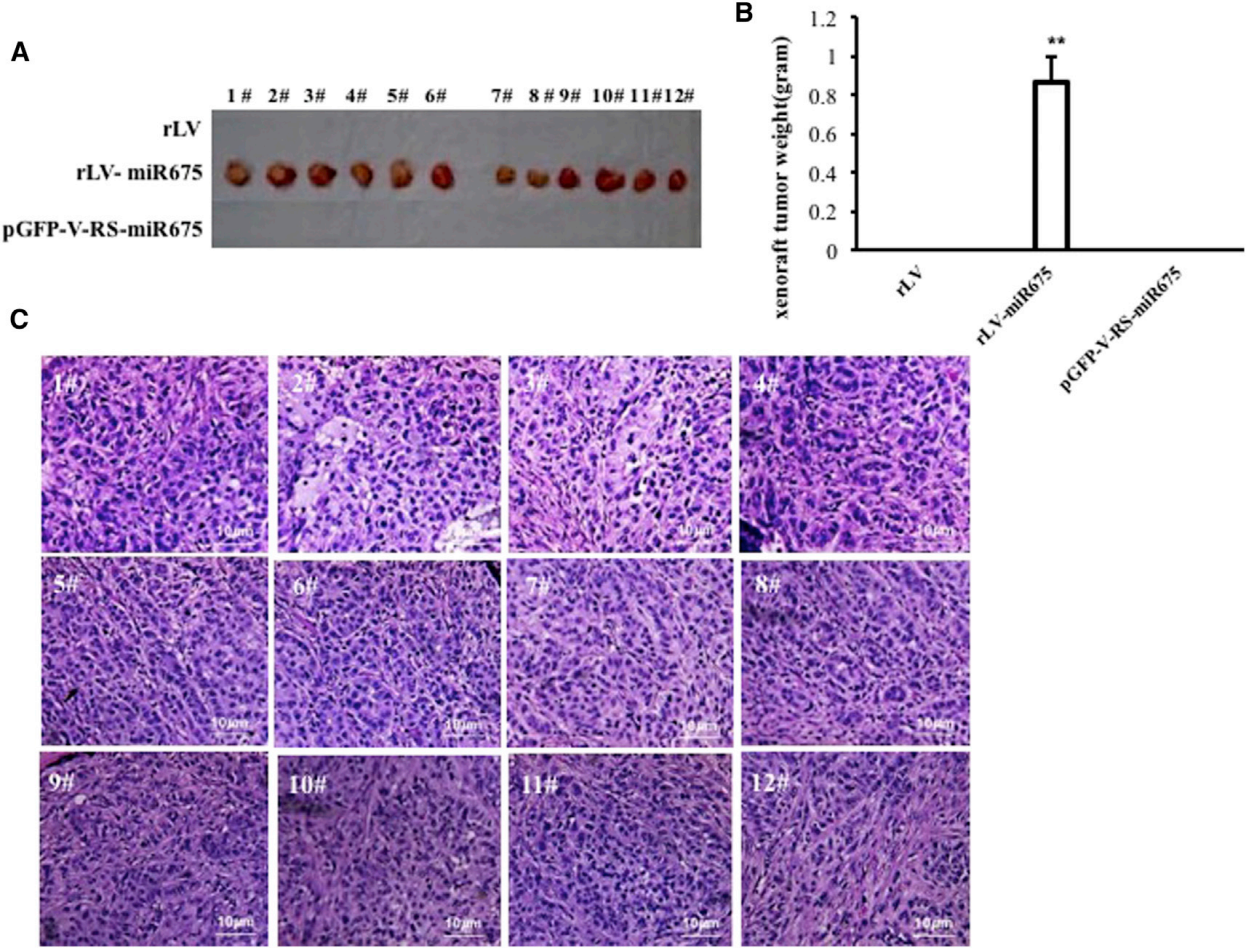
Tumorigenesis Test *In Vivo* (A) The mice were stratified and the tumors were recovered; the photography shows xenograft tumor in the three groups (indicated at left, n = 12). (B) The wet weight of each tumor was determined for each mouse. Each value was presented as mean ± SEM. (C) A portion of each tumor was fixed in 4% paraformaldehyde and embedded in paraffin for histological H&E staining (original magnification ×100).

To further determine whether miR675 promotes malignant transformation of HBMMSCs *in vivo*, the HBMMSC cell lines from three groups (rLV group, rLV-miR675 group, and rLV-Gas9-miR675 group) were injected subcutaneously at armpit into athymic BALB/c mice. The findings showed that miR675-overexpressing HBMMSC cells were transformed into xenograft tumors (0.85975 ± 0.1406 g, n = 12), whereas the other groups did not appear in xenograft tumors at all (p < 0.01) (Figures 2A and 2B). Furthermore, pathological examination (H&E staining) of the xenografts revealed a poorly differentiated tumor cells (Figure 2C). Taken together, miR675 promotes the malignant transformation and growth of HBMMSCs.

### miR675 Enhances the Expression of P62

**Figure 3:**
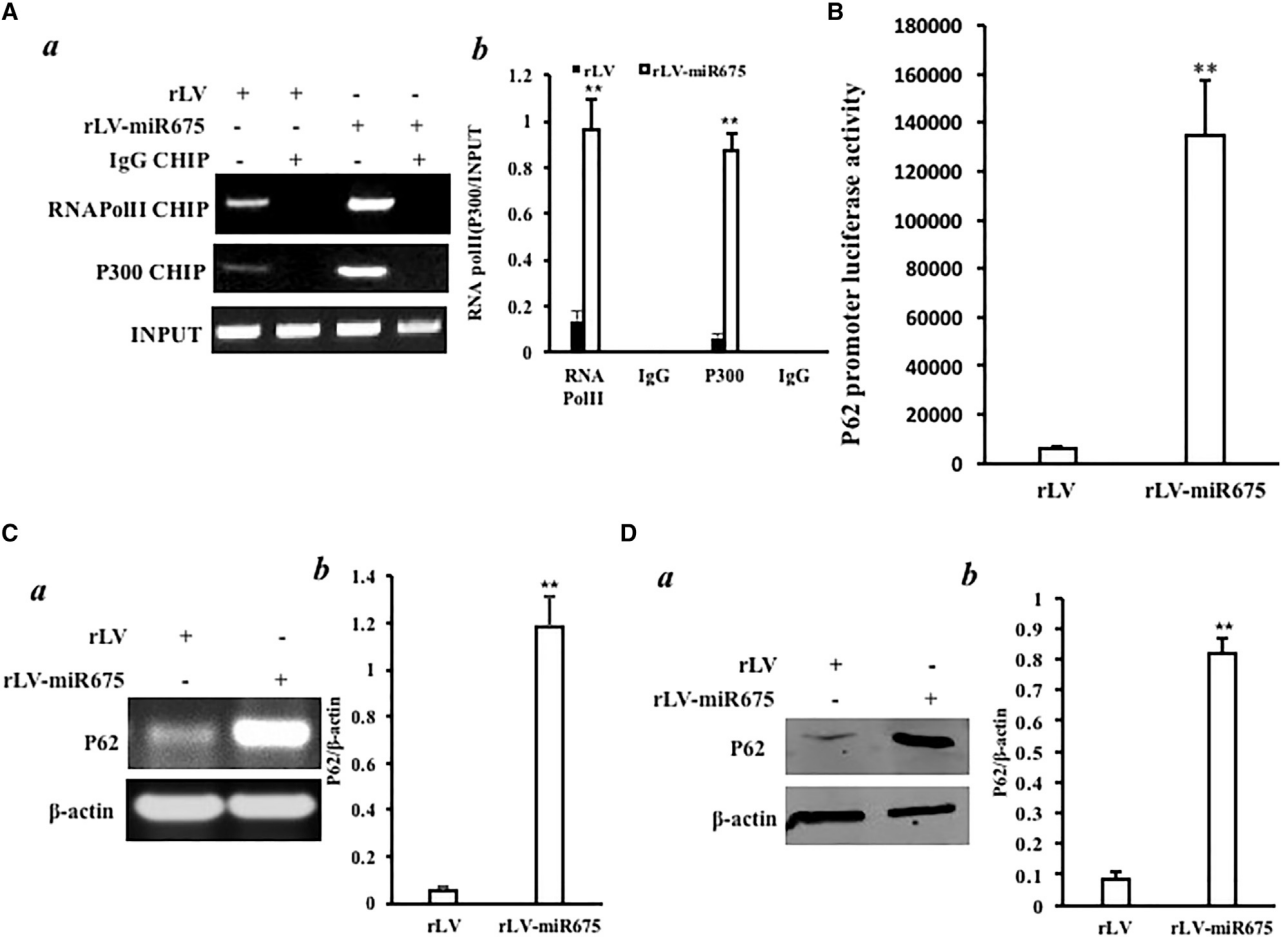
miR675 Enhances the Expression of P62 in the Human Mesenchymal Stem Cells Infected with rLV and rLV-miR675, Respectively (A) (a) Chromatin immunoprecipitation (ChIP) with anti-P300 and Pol II followed by PCR with P62 promoter primers. IgG ChIP was the negative control. P62 promoter DNA was the INPUT. (b) The quantitative analysis of ChIP. (B) The activity assay of P62 promoter luciferase reporter gene. (C) (a) P62 expression analysis by RT-PCR P62 in the human mesenchymal stem cells infected with rLV and rLV-miR675, respectively. β-actin was the internal control. (b) The quantitative analysis of RT-PCR. (D) (a) P62 expression analysis by western blotting with anti-P62 in the human mesenchymal stem cells infected with rLV and rLV-miR675, respectively. β-actin was the internal control. (b) The gray scan analysis of positive bands of western blotting.

Given that the *P62* gene encodes a multifunctional protein as a scaffolding-adaptor that regulates activation of several signaling pathways, we considered whether miR675 affects the expression of P62. At the first time, chromatin immunoprecipitation (ChIP) results showed that excessive miR675 enhanced the binding of CREB and P300 to the P62 promoter region in the HBMMSCs (Figure 3A a and b). Moreover, excessive miR675 enhanced p62 promoter luciferase activity compared to control (6,309.667 ± 555.29 versus 134,460 ± 23,297.39; p = 0.0057 < 0.01) in the HBMMSCs (Figure 3B). Ultimately, RT-PCR findings showed that excessive miR675 enhanced the expression of *P62* on the transcriptional level (Figure 3C a and b), and western blotting findings showed that excessive miR675 enhanced *P62* on the translational level (Figure 3D a and b).Taken together, excessive miR675 enhanced the expression of P62 in the HBMMSCs.

### miR675 Decreases the Interaction among hMSH6, H3k36me3, and Skp2, Dependent on P62

**Figure 4:**
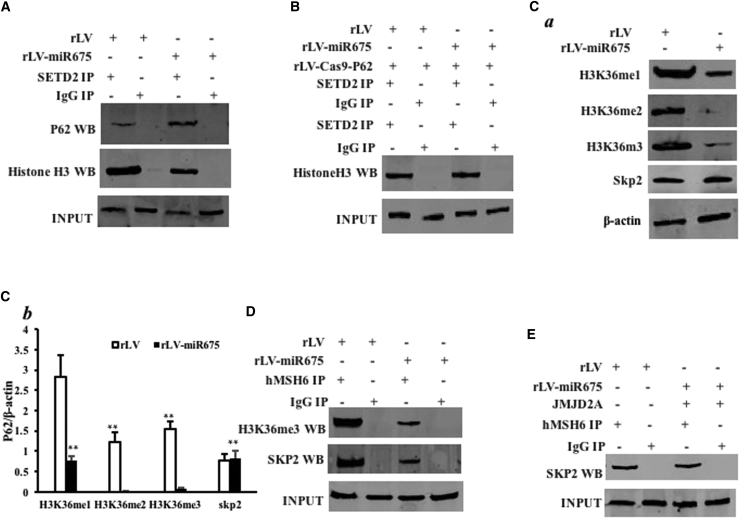
miR675 Affects the Interaction among hMSH6, H3k36me3, and SKP2 in the Human Mesenchymal Stem Cells Infected with rLV and rLV-miR675, Respectively (A) Anti-STED2 co-immunoprecipitation (coIP) followed by western blotting with anti-P62 and anti-histone 3. IgG IP was the negative control. INPUT refers to western blotting with anti-STED2. (B) Anti-SETD2 coIP followed by western blotting with anti-histone 3. IgG IP was the negative control. INPUT refers to western blotting with anti-SETD2. (C) (a) Western blotting with anti-H3K36me1, anti-H3K36me2, and anti-H3K36me1. β-actin was the internal control. (b) The gray scan analysis of positive bands of western blotting. (D) Anti-hMSH6 coIP followed by western blotting with anti-H3K36me3 and anti-SKP2. IgG IP was the negative control. INPUT refers to western blotting with anti-hMSH6. (E) Anti-hMSH6 coIP followed by western blotting with anti-SKP2 in the human mesenchymal stem cells, including the rLV and rLV-miR675 plus pCMV6-AC-GFP-JMJD2A groups. IgG IP was the negative control. INPUT refers to western blotting with anti-hMSH6.

Given that the ternary complex hMSH6-H3k36me3-Skp2 plays an important role in the damaged DNA and aging protein repair, we wondered whether miR675 influences the formation of the complex via P62, which regulates and controls the modification of three methylations on histone H3 36th lysine. As shown in Figure 4A, the overexpression of miR675 enhanced the interaction between SETD2 and P62, and it decreased the interaction between SETD2 and histone H3. However, when P62 was knocked down, the overexpression of miR675 did not significantly alter the interaction between SETD2 and P62 and the interaction between SETD2 and histone H3 compared to control (Figure 4B). Furthermore, the overexpression of miR675 decreased the levels of H3K36me1, H3K36me2, and H3K36me3 compared to control. However, the expression of Skp2 did not significantly vary between the rLV and rLV-miR675 groups (Figure 4C a and b). Moreover, the overexpression of miR675 inhibited the interaction between hMSH6 and H3K36me3 and between hMSH6 and Skp2 compared to control (Figure 4D). In particular, the overexpression of miR675 did not alter the interaction between hMSH6 and Skp2 when these cells were transfected with pGFP-V-RS-KDM4A (knockdown of KDM4A, H3K36 demethylase) (Figure 4E), suggesting that H3K36me3 promotes the interaction of hMSH6 with Skp2 in the HBMMSCs. Together, our results underline that miR675 inhibits the level of H3K36me3 and reduces the ternary complex of hMSH6-H3k36me3-Skp2 in human HBMMSCs.

### miR675 Reduces the hMSH6-H3k36me3-Skp2 Ternary Complex Loading onto the Site of DNA Mismatch and Increased the H3.3 on the DNA Mismatch Region

**Figure 5:**
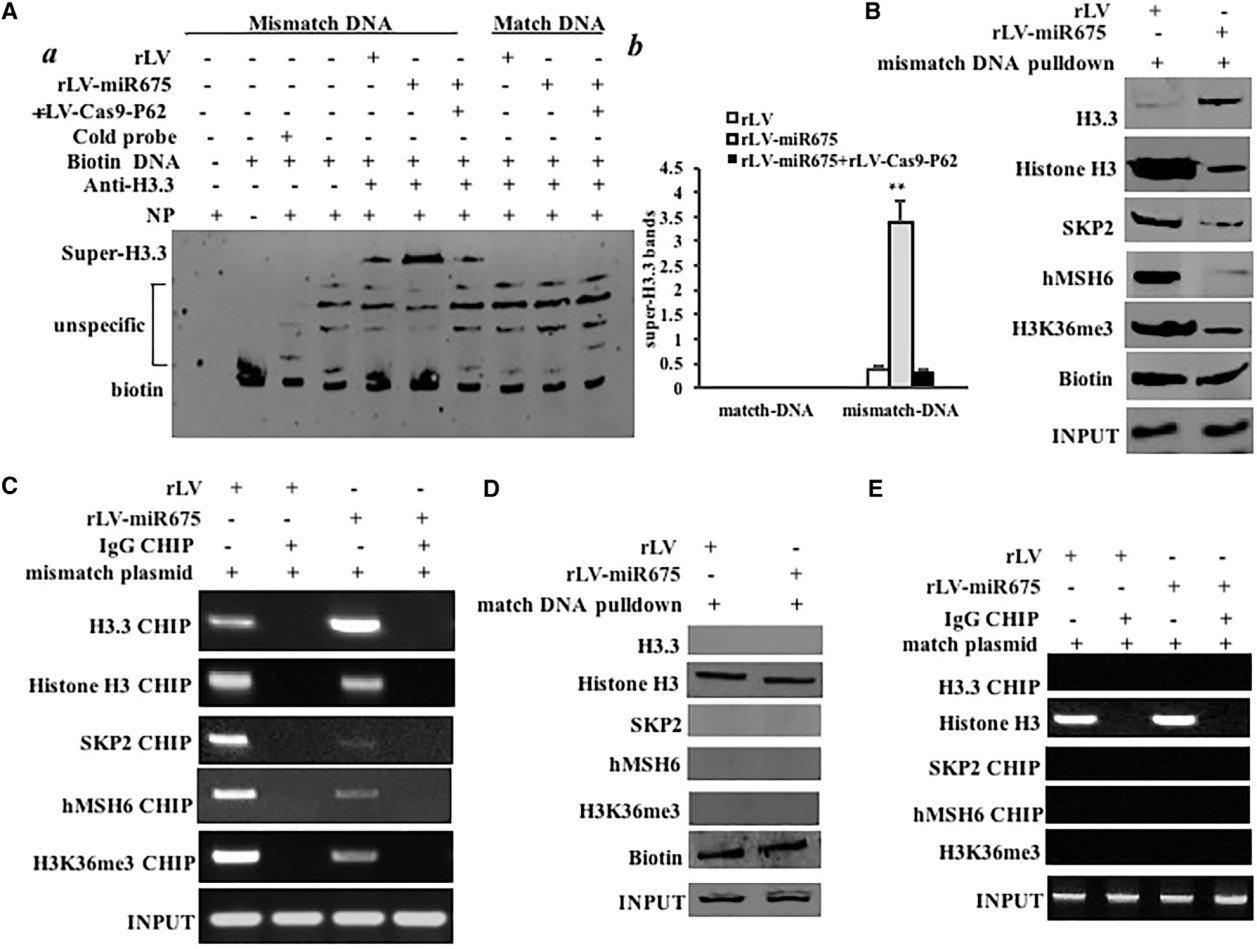
miR675 Delays the hMSH6-H3k36me3-Skp2 Ternary Complex Occupancy on the Mismatch DNA in the Human Mesenchymal Stem Cells Infected with rLV and rLV-miR675, Respectively (A) (a) Super-EMSA (gel shift) with biotin-DNA probe (including mismatch and match DNA double strands) and anti-histone H3.3 antibody. The intensity of the band was examined by western blotting with anti-biotin. (b) The gray scan analysis of positive bands of super-EMSA. (B) Biotin-mismatch probe pull-down followed by western blotting with anti-histone 3.3, anti-histone 3, anti-SKP2, anti-hMSH6, and anti-H3K36me3. Biotin was the INPUT and histone was the internal control. (C) Chromatin immunoprecipitation (ChIP) with anti-histone 3.3, anti-histone H3, anti-SKP2, anti-hMSH6, and anti-H3K36me3 followed by PCR with damaged DNA primers. IgG ChIP was the negative control and damaged DNA was the INPUT. (D) Biotin-match double DNA probe pull-down followed by western blotting with anti-histone H3.3, anti-histone H3, anti-SKP2, anti-hMSH6, and anti-H3K36me3. Biotin was the INPUT and histone was the internal control. (E) ChIP with anti-histone 3.3, anti-histone H3, anti-SKP2, anti-hMSH6, and anti-H3K36me3 followed by PCR with match double DNA primers. IgG ChIP was the negative control and match double DNA was the INPUT.

To address whether miR675 impacted DNA damage repair in HBMMSCs, we constructed a DNA mismatch plasmid (EcoRI mismatch), which could be repaired after the plasmid was transfected and integrated into the chromosome. After transfection with the plasmid, we performed anti-H3.3 super-electrophoretic mobility shift assay (EMSA) with biotin-mismatch DNA and biotin-match DNA. Our results showed that overexpression of miR675 increased the binding of histone H3.3 to mismatch DNA, and P62 knockdown abrogated the action of miR675. However, the binding of histone H3.3 to match DNA was not discovered in these HBMMSCs (Figure 5A a and b). Biotin-mismatch DNA pull-down findings showed that the overexpression of miR675 increased the binding of histone H3.3 to mismatch DNA and decreased the binding of mismatch DNA to histone H3, SKP2, H3k36me3, and hMSH6 (Figure 5B). Furthermore, ChIP experiments revealed that overexpression of miR675 promoted the loading of histone H3.3 onto mismatch DNA and inhibited the loading of histone H3, Skp2, H3k36me3, and hMSH6 onto the mismatch DNA (Figure 5C). Moreover, biotin-match DNA pull-down findings showed match DNA binds to histone H3, but not to histone 3.3, Skp2, H3k36me3, and hMSH6 in these HBMMSCs, and miR675 did not alter this binding ability (Figure 5D). ChIP findings showed the loading of histone H3 onto the match DNA, but not histone 3.3, Skp2, H3K36me3, and hMSH6 in these HBMMSCs, and miR675 did not alter the loading of histone H3 onto the match DNA (Figure 5E). These results, taken together, suggest that the miR675 made concerted efforts to prevent the hMSH6-H3k36me3-Skp2 ternary complex from occupancy on the site of DNA mismatch and increase the histone H3.3 on the DNA mismatch region.

### miR675 Inhibits DNA MMR, and It Blocks Ubiquitination and Replacement of Aging Histone H3.3 in Response to DNA Damage

**Figure 6:**
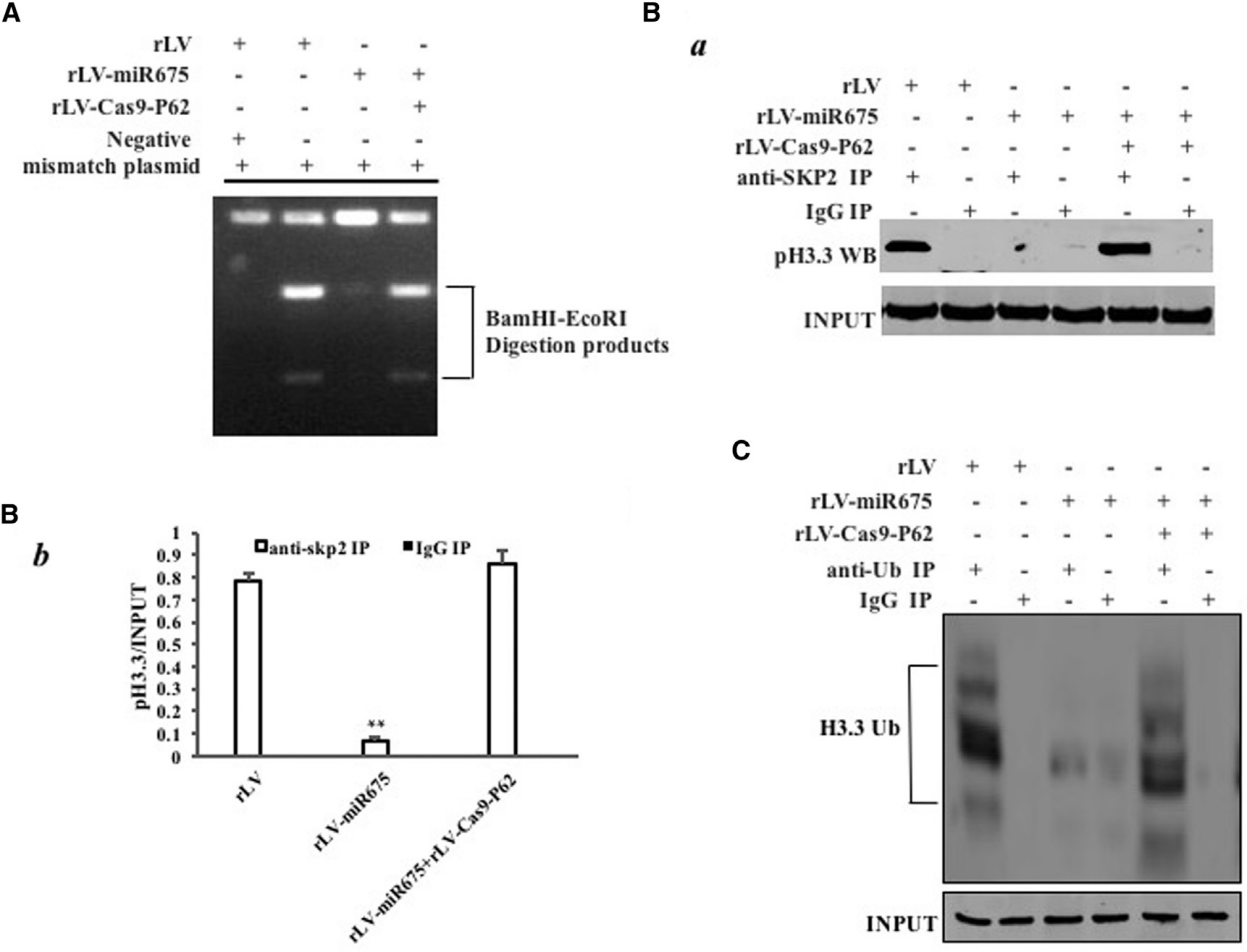
miR675 Inhibits DNA Damage Repair and Decreases Aging Histone H3.3 Degradation in the Human Mesenchymal Stem Cells Infected with rLV, rLV-miR675, and rLV-miR675 Plus rLV-Cas9-P62, Respectively (A) Restriction endonuclease analysis with BamHI and EcoRI for plasmid DNA injury repair. (B) (a) Anti-SKP2 coIP followed by western blotting with anti-histone 3.3. IgG IP was the negative control. INPUT refers to western blotting with anti-histone H3.3. (b) The gray scan analysis of positive bands. (C) Anti-Ub coIP followed by western blotting with anti-histone H3.3. IgG IP was the negative control. INPUT refers to western blotting with β-actin.

To reveal whether miR675 inhibits DNA MMR, we carried out several assays *in vitro* and *in vivo*. We extracted the DNA from these HBMMSCs. Then a reaction *in vitro* using mismatch plasmid mixing with nuclear extract (then PCR reaction using mismatch plasmid primers) was carried out, followed by restriction endonuclease analysis with BamHI and EcoRI for DNA MMR. As shown in Figure 6A, overexpression of miR675 significantly reduced the BamHI and EcoRI restriction product, whereas knockout of P62 significantly abrogated this action. To further examine miR675’s effect on histone H3.3, we performed co-immunoprecipitation (coIP) experiments. Overexpression of miR675 impedes the interaction between SKP2 and histone H3.3, however, knockout of P62 significantly abrogated this action (Figure 6B a and b). Strikingly, overexpression of miR675 decreased the ubiquitination of histone H3.3 in these HBMMSCs compared to the control (Figure 6C). These observations suggest that miR675 impedes DNA MMR and decreases the degradation of histone H3.3.

### miR675 Triggers Microsatellite Instability and Abnormal Expression of Genes

**Figure 7:**
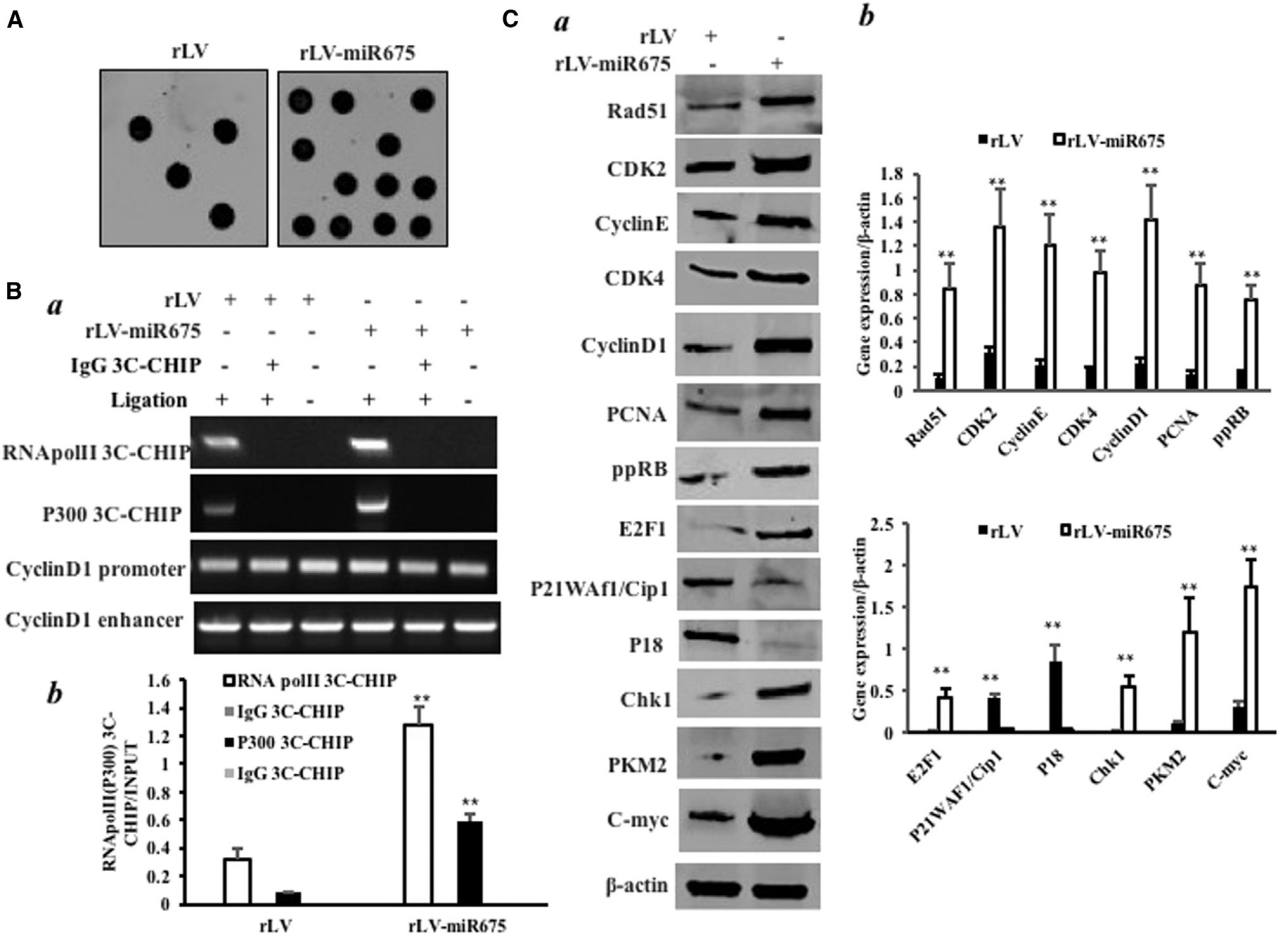
miR675 Triggers MSI and Abnormal Gene Expression in the Human Mesenchymal Stem Cells Infected with rLV and rLV-miR675, Respectively (A) Microsatellite instability (MSI) analysis through dot blot (slot blot) using various biotin-labeling MSI probes (biotin-MSIs). (B) (a) Chromosome conformation capture (3C)-chromatin immunoprecipitation (ChIP) with anti-P300 and anti-Pol II. The chromatin was cross-linked, digested with restriction enzymes, and ligated under conditions that favor intramolecular ligation. Immediately after ligation, the chromatin was immunoprecipitated using an antibody (anti-P300, anti-Pol II) against the protein of interest. Thereafter, the cross-links were reversed and the DNA was purified further. The PCR anlysis was applied for detecting CyclinD1 promoter-enhancer coupling product using CyclinD1 promoter and enhancer primers. The CyclinD1 promoter and enhancer was the INPUT. (b) The quantitative analysis of ChIP-3C. (C) (a) Western blotting with anti-Rad51, anti-CDK2, anti-CyclinE, anti-CDK4, anti-CyclinD1, anti-PCNA, anti-ppRB, anti-E2F1, anti-P18, anti-P21, anti-PKM2, anti-c-Myc, and anti-Chk1. β-actin was the internal control. (b) The gray scan analysis of positive bands of western blotting.

Cells lacking the H3K36 tri-methyltransferase SETD2 display microsatellite instability (MSI) and an elevated spontaneous mutation frequency in cells. We performed an MSI assay through dot blot (slot blot) using various biotin-labeling MSI probes (biotin-MSIs) in these HBMMSCs. As shown in Figure 7A, overexpression of miR675 increased the MSI compared to the control group. Moreover, the overexpression of miR675 increased the formation of CyclinD1 promoter-enhancer DNA loop entering RNA polymerase II (Pol II) and P300 (Figure 7B a and b). Furthermore, miR675 increased the expression of Rad51, CDK2, CyclinE, CDK4, CyclinD1, PCNA, ppRB, E2F1, Chk1, PKM2, and c-Myc, and it decreased the expression of P18 and P21/WAF1/Cip1 in these HBMMSCs compared to the control (Figure 7C a and b). Together, these observations suggest that miR675 triggers MSI and abnormal expression of cell cycle-related genes, e.g., Rad51, CDK2, CyclinE, CDK4, CyclinD1, PCNA, ppRB, E2F1, PKM2, c-Myc, Chk1, P21 (WAF-Cip1), and P18.

### P62 Depletion Abrogates the Oncogenic Function of miR675

**Figure 8:**
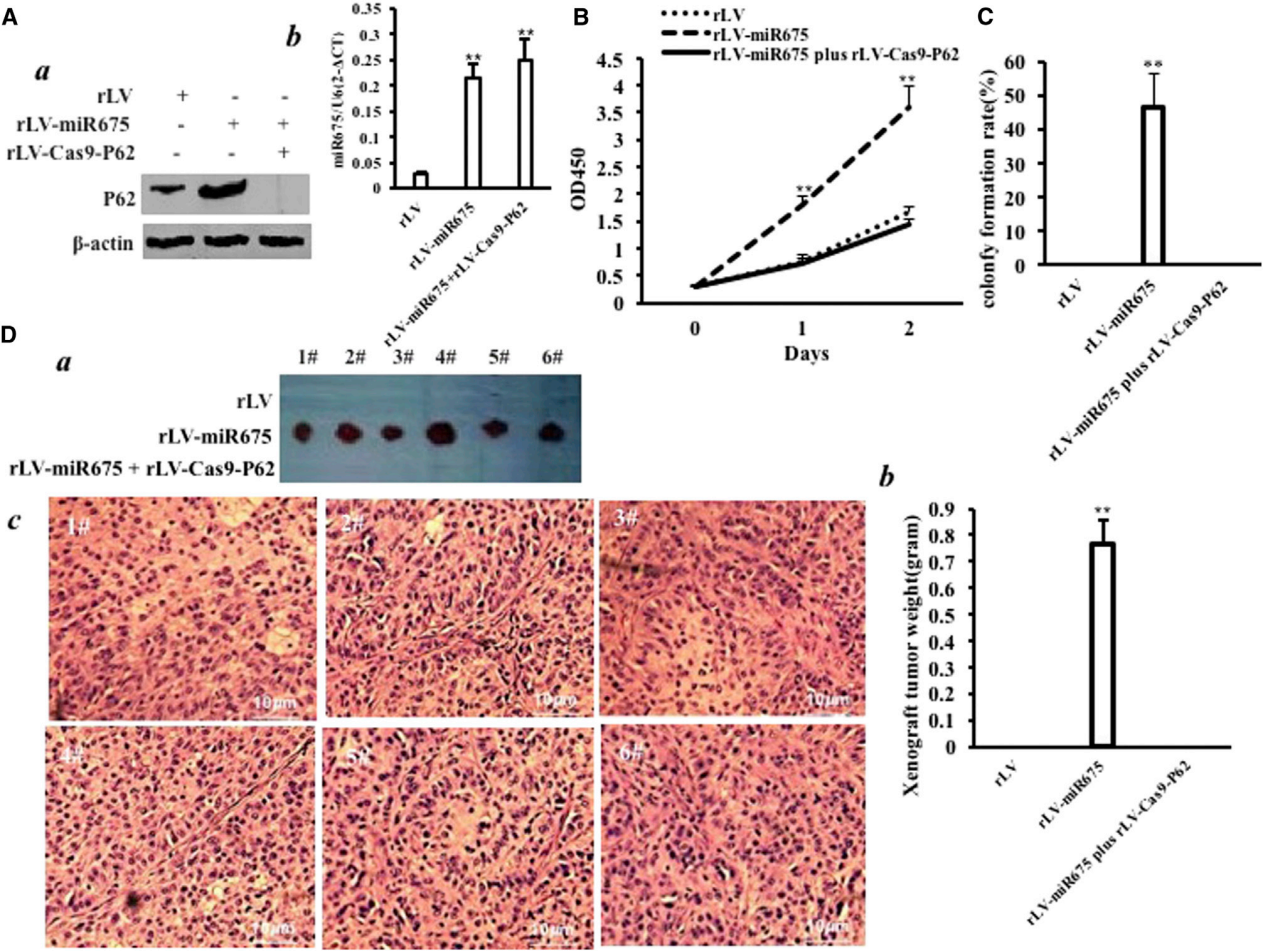
The Rescue Experiment of the Carcinogenesis Effect of miR675 P62 knockdown abrogated the oncogenic function of miR675 in the human mesenchymal stem cells infected with rLV, rLV-miR675, and rLV-miR675 plus rLV-Cas9-P62, respectively. (A) (a) The western blotting analysis with anti-P62. β-actin was the internal control. (b) The real-time RT-PCR analysis for mature miR675. U6 was the internal control. (B) Cell growth assay using CCK8. Each value was presented as mean ± SEM. (C) Cell soft agar colony formation assay. (D) Tumorigenesis test *in vivo*. (a) The mice were stratified and the tumors were recovered. (b) The wet weight of each tumor was determined for each mouse. Each value was presented as mean ± SEM. (c) A portion of each tumor was fixed in 4% paraformaldehyde and embedded in paraffin for histological H&E staining.

To further demonstrate whether the oncogenic effect of miR675 is dependent on P62, we conducted a rescue experiment. We first constructed the stable HBMMSC cell lines infected with rLV, rLV-miR675, and rLV-miR675 plus rLV-Cas9-P62. P62 was overexpressed in the rLV-miR675 group and decreased in the rLV-mir675 plus rLV-Cas9-P62 group compared to the rLV group (Figure 8A a). Mature miR675 was significantly increased in the rLV-miR675 group and the rLV-mir675 plus rLV-Cas9-P62 group compared to the rLV group, respectively (Figure 8A b). Cell growth was more rapid in the group of rLV-miR675 compared to the control. Intriguingly, the action of miR675 was abrogated when p62 was knocked out in these HBMMSCs (Figure 8B). As shown in Figure 8C, the colony formation efficiency rate was 46.4% ± 9.93% in the group of rLV-mir675, whereas colony formation was not exhibited in the rLV and rLV-mir675 plus rLV-Cas9-P62 groups. Furthermore, the xenografts were only produced in the group of rLV-miR675 (0.763 ± 0.093 g, n = 6), whereas xenograft tumors were not produced in the rLV and rLV-mir675 plus rLV-Cas9-P62 groups (Figure 8D a and b). Moreover, the xenografts showed poorly differentiated tumor cells (Figure 8D c). Taken together, the overexpression of miR675 causes the malignant transformation of HBMMSCs, and the oncogenic activity of miR675 may be decided by P62 at least partly.

## Discussion

**Figure 9:**
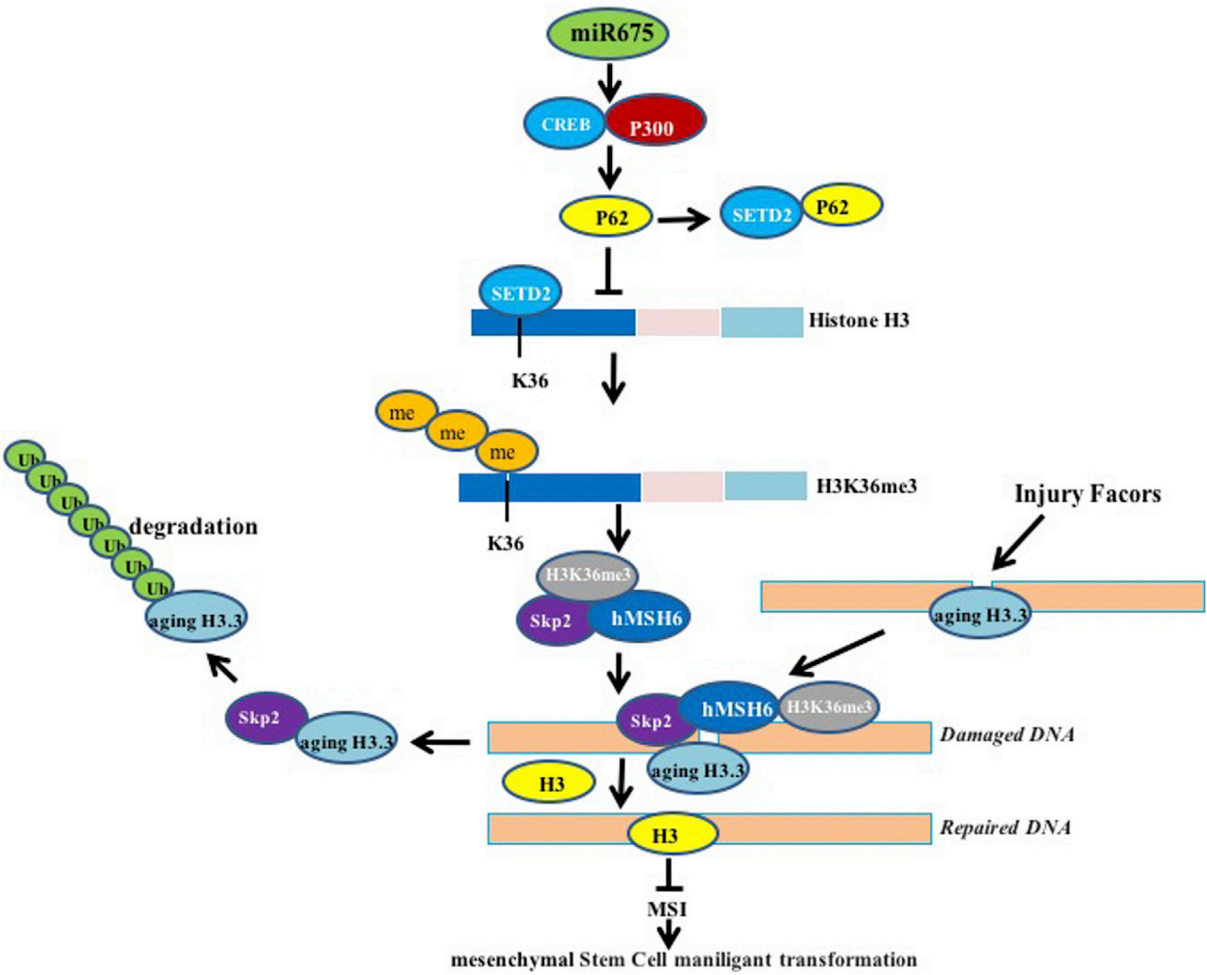
The Schematic Illustrates a Model of the Differentiation of Human Mesenchymal Stem Cells into Cancer Cells via miR675 Oncogenic miR675 promotes the interaction between CREB and P300, which leads to the high expression of P62. That P62 competes with STED2 to bind histone H3 greatly reduces the STED2-binding capacity with substrate histone H3, triggering a reduction of three methylations on histone H3 36th lysine (H3K36me3); thereby, the H3K36me3-hMSH6-SKP2 tri-complex is decreased. Meanwhile, the ternary complex occupancy capacity on chromosome is absolutely reduced, preventing normal DNA repair. By virtue of the reductive degradation ability of SKP2 for aging histone H3.3 bound to damaged DNA, the aging histone H3.3 repair is delayed and eliminated. That the damaged DNA escaped repair can lead to the abnormal expression of some cell cycle- and metabolism-related genes, causing the human mesenchymal stem cell malignant transformation.

At the present, the functions and regulatory mechanism of miR675 have not been elucidated. To our knowledge, this paper might be the first to demonstrate that miR675 promotes hMSC malignant transformation. As shown in Figure 9, we provide evidence that miR675 promotes the malignant proliferation and transformation of MSCs *in vitro* and *in vivo*. Strikingly, our results also reveal that P62 is required for miR675 oncogenic action.

First, accumulating evidence indicates that miR-675 was found to be upregulated in human cancer^22^ and promotes the proliferative and growth capacity of cancer.^7^ Our present results are consistent with these reports, and they provide novel evidence for an active role of miR675 in promoting MSC malignant formation and growth. This evidence is based on results from two parallel sets of experiments: (1) miR675 promotes the proliferation of MSCs, and, (2) in particular, miR675 accelerates the malignant transformation of MSCs, including colony formation ability and tumorigenesis, *in vivo*.

Our findings in this study provide novel evidence for an active role of P62 in the miR675-mediated promotion of malignant formation. This assertion is based on several observations: (1) miR675 enhanced the expression of P62 in the HBMMSCs; (2) miR675 inhibited the level of SETD2 (SETD2 protein is specific for lysine-36 of histone H3, which is associated with active chromatin through enhancing P62), which decreases the H3K36me3; (3) miR675 decreases the interaction among hMSH6, H3k36me3, and Skp2, dependent on P62; and (4) although the overexpression of miR675 causes the malignant transformation of HBMMSCs, excessive P62 depletion abrogates the oncogenic function of miR675. p62 acts through the modulation of metabolism in the tumor stroma.^23^ P62 gene encodes a multifunctional protein that binds ubiquitin^24^ and promotes tumorigenesis through some signaling pathway.^12^, ^25^, ^26^ Moreover, P62 regulates and controls autophagy in human tumors.^14^, ^27^ Furthermore, the dysregulation of P62 reduces the ability to repair DNA.^28^, ^29^

It is worth mentioning that excessive miR675 causes epigenetic deregulation. Methylated histone readers are critical for chromatin dynamics, transcription, and DNA repair. H3K36me3 binds to chromatin of active genes in an MMR-dependent manner.^30^, ^31^ H3K36 methylation promotes DNA damage insults.^32^, ^33^ Importantly, histone H3 variants are related to DNA MMR.^34^, ^35^ Moreover, the dynamics of histone H3 variants trigger heterochromatin organization.^36^

Our results suggest that miR675 impedes DNA MMR. This assertion is based on several observations: (1) miR675 inhibited the level of H3K36me3 through inhibiting SETD2, (2) miR675 decreased the interaction between hMSH6 and H3k36me3, and (3) miR675 reduced the ternary complex of hMSH6-H3k36me3-Skp2 in human HBMMSCs. Research shows that MMR ensures replication fidelity by correcting mismatches generated during DNA replication.^37^, ^38^ DNA MMR and direct reversal of damage (DRD) are two repair mechanisms that help in the removal of faulty base pairs and alkyl adduct formation, respectively, to avoid long-term effects of toxicity, tumorigenesis, and mutagenesis.^39^

Another significant finding is that miR675 inhibits aging histone H3.3 replacement on mismatch DNA and then blocks the DNA damage repair. This is based on several results of mR675 overexpression in MSCs: (1) miR675 decreased the interaction among hMSH6, H3k36me3, and SKP2 and reduced the complex of hMSH6-H3k36me3-SKP2; (2) furthermore, miR675 reduced the hMSH6-H3k36me3-SKP2 ternary complex loading onto the site of DNA damage; (3) thereby, miR675 reduced the interaction between SKP2 and aging histone 3.3; and (4) miR675 blocked the degradation of the aging histone H3.3, which bound to damaged DNA.

In particular, our results also show that miR675 triggers MSI. Researchers indicated that DNA MMR may lead to MSI and triggers the abnormal expression of genes.^40^, ^41^ In this study, our results showed that miR675 enhances the abnormal expression of cell cycle-related genes, e.g., Rad51, CDK2, CyclinE, CDK4, CyclinD1, PCNA, ppRB, E2F1, PKM2, c-Myc, and Chk1, and it inhibits the expression of P21/WAF/Cip1 and P18 through MSI.

Finally, the function of miR675 in MSCs should be further explored. Many questions remained about the function of miR675. For example, what causes the strong oncogenic action of miR675? How does miR675 cooperate with P62? Does miR675 enhance malignant differentiation of MSCs? Does miR675 regulate a series of molecular events for the malignant differentiation of MSCs? Answering these questions will help to understand the mechanisms about the malignant differentiation of MSCs.

In summary, our present data indicated that miR675 promotes hMSC malignant progression through epigenetic hallmarks, with diagnostic and prognostic implications. We believe miR675 will be a key target for the development and progression of cancer stem cells.

## Materials and Methods

### Cell Lines and Lentivirus

Normal HBMMSCs were purchased from ATCC (ATCC PCS-500-012; Manassas, VA). HBMMSCs were maintained in DMEM supplemented with 10% heat-inactivated fetal bovine serum (Gibco BRL Life Technologies) in a humidified atmosphere of 5% CO_2_ incubator at 37°C. Lentivirus rLV-Green, rLV-Green-miR675, and rLV-Cas9 were purchased from Wu Han Viraltherapy Technologies.

### Antibodies and Primers

Standard western immunoblotting procedures were used with the following antibodies: anti-H3K36me1 (Abcam), anti-H3K36me2 (Abcam), anti-H3K36me3 (Abcam), anti-SETD2 (Santa Cruz Biotechnology), anti-hMSH6 (Santa Cruz Biotechnology), anti-histone 3.3, anti-histone 3, anti-SKP2 (Santa Cruz Biotechnology), anti-biotin (Santa Cruz Biotechnology), anti-Ub (Santa Cruz Biotechnology), anti-P300 (Abcam), anti-RNA Pol II (Abcam), anti-CyclinE (Santa Cruz Biotechnology), anti-CDK4 (Santa Cruz Biotechnology), anti-CyclinD1 (Abcam), anti-PCNA (Abcam), anti-ppRB (Abcam), anti-E2F1 (Abcam), anti-P18 (Abcam), anti-P21/WAF1/Cip1 (Santa Cruz Biotechnology), anti-PKM2, anti-c-Myc (Santa Cruz Biotechnology), anti-Chk1 (Abcam), anti-P62 (Abcam), anti-KDM4A (Abcam), and anti-β-actin (Abcam).

The primers were as follows: P62: P1, 5′-GCAGTATCCCAAGTTCAATT-3′; P2, 5′-TGGGAACAGGTGGTGGAGGA-3′; P62 promoter: P1, 5′-GATCATTCACACCTGTGGAC-3′; P2, 5′-GGACGAGTGGTCACCCTCTG-3′; pre-miR-675: P1, 5′-CCCAGGGTCTGGTGCGGAGA-3′; P2, 5′-CCCAGGGGCTGAGCGGTGAG-3; mature mi675: P1, 5′-TGGTGCGGAGAGGGCCACAGUG-3′; U6 primer: P1, 5′-GCTTCGGCAGCACATATACT-3′; P2, 5′-GGAACGCTTCACGAATTTGC-3′; and β-actin: P1, 5′-CTTCCTTCCTGGGCATGGAG-3′; P2, 5′-TGGAGGGGCCGGACTGGTCA-3′.

### Western Blotting

Western blotting was carried out according to methodology as previously described.^42^ Briefly, samples were separated on an SDS-PAGE and incubated with antibody. Secondary antibodies were as follows: anti-rabbit immunoglobulin G (IgG) and anti-mouse IgG (GE Healthcare). Signals were visualized by an enhanced chemiluminescence (ECL) system (Amersham), according to the manufacturer’s instructions.

### coIP

coIP was carried out according to methodology as previously described.^42^

### DNA Pull-down

DNA pull-down was carried out according to methodology as previously described.^42^ Briefly, DNA-bound proteins were collected for incubation with streptavidin-agarose resin for 4 hr. Following washings of the resin-bound complex with binding buffer, the samples were subjected to SDS-PAGE for western blotting analysis with related antibodies.

### Super-EMSA (Gel Shift)

Super-EMSA was carried out according to methodology as previously described.^43^

### ChIP Assay

ChIP assay was carried out according to methodology as previously described.^43^ Briefly, cells were cross-linked with 1% (v/v) formaldehyde (Sigma) for 10 min at room temperature. Chromatin extracts were immunoprecipitated with specific antibody on Protein-A/G-Sepharose beads. After washing and de-cross-linking, the ChIP DNA was detected by PCR.

### Chromosome Conformation Capture-ChIP Assay (ChIP-3C/ChIP-Loop Assays)

ChIP-chromosome conformation capture (3C) was carried out according to methodology as previously described.^43^ Briefly, antibody-specific immunoprecipitated chromatin was obtained as described above for ChIP assays. Samples were then incubated with restriction enzyme. After digestion and de-cross-linking, the ChIP-3C material was detected by PCR with specific primers.

### MSI Detection through Dot Blot (Slot Blot)

Slot blot was carried out according to methodology as previously described.^44^

### Cell Proliferation CCK8 Assay

CCK8 was carried out according to methodology as previously described.^45^ The cell proliferation reagent CCK8 was purchased from Roche, and the operation was performed according to the manufacturer’s instructions.

### Soft Agar Colony Formation Assay

Soft agar colony formation assay was carried out according to methodology as previously described.^45^ Briefly, cells were plated on a double-layer soft agar plate and incubated for 21 days. Soft agar colonies were stained with Crystal Violet (Henan Tianfu Chemical).

### Xenograft Transplantation *In Vivo*

Tumorigenesis *in vivo* was carried out according to methodology as previously described.^46^ Briefly, the athymic BALB/c mice were injected at the armpit area subcutaneously with HBMMSCs. The use of mice for this work was reviewed and approved by the institutional animal care and use committee in accordance with China national institutes of health guidelines.

## Author Contributions

D.L. conceived the study and participated in the study design, performance, coordination, and manuscript writing. Y.L., S.S., X.J., Q.M., C.W., X.L., Y.Y., X.X., Q.Z., L.W., H.P., X.G., and T.L. performed the research. All authors read and approved the final manuscript.

## Conflicts of Interest

The authors declare no competing interests.

## Supplemental Information

Document S1. Figures S1 and S2

Document S2. Article plus Supplemental Information
