# Supplementary material for: miR675 Accelerates Malignant Transformation of Mesenchymal Stem Cells by Blocking DNA Mismatch Repair
Source: Mol Ther Nucleic Acids. 2018 Nov 24;14:171–83. doi: 10.1016/j.omtn.2018.11.010 (PMC6307386; doi:10.1016/j.omtn.2018.11.010)
Supplement: Document S1. Figures S1 and S2 [file mmc1.pdf]

OMTN, Volume *14*

Supplemental Information

miR675 Accelerates Malignant Transformation of Mesenchymal Stem Cells by Blocking DNA Mismatch Repair

Yanan Lu, Shuting Song, Xiaoxue Jiang, Qiuyu Meng, Chen Wang, Xiaonan Li, Yuxin Yang, Xiaoru Xin, Qidi Zheng, Liyan Wang, Hu Pu, Xin Gui, Tianming Li, and Dongdong Lu

Figure S1

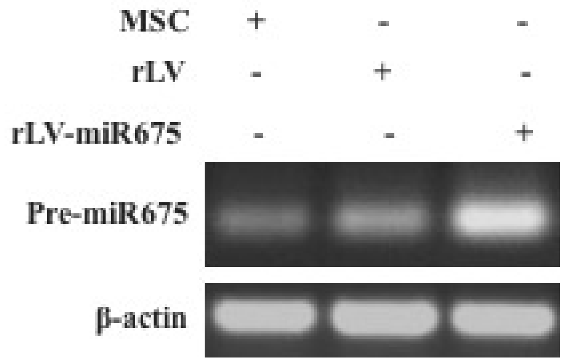

Figure S2

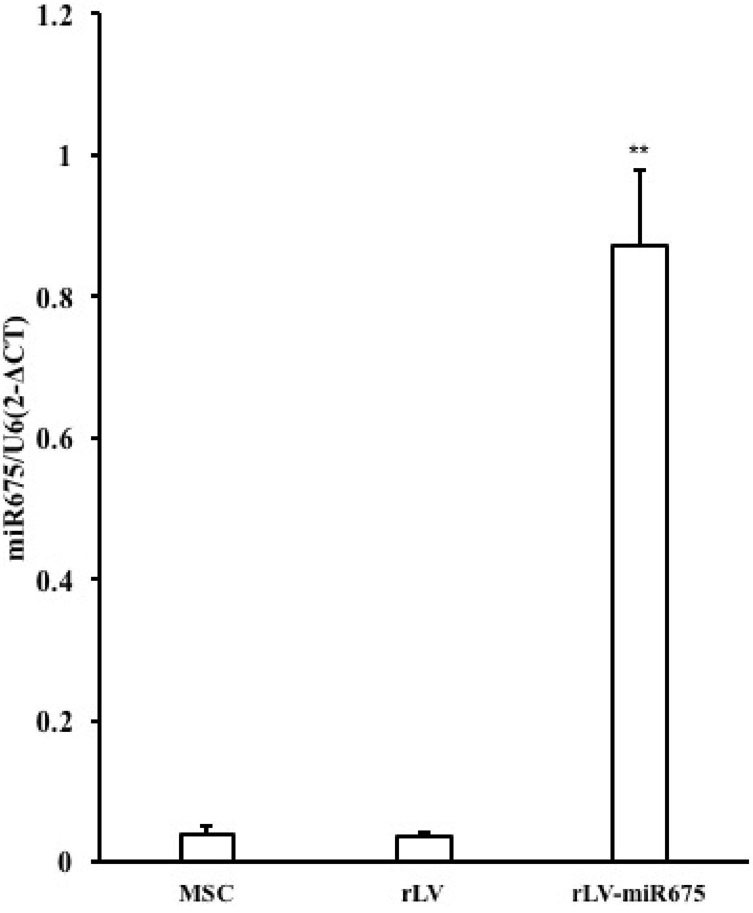

Supplemental FIGURE LEGENDS

FigureS1 RT-PCR analysis of pre-miR675 in the human mesenchymal stem cells (HBMMSCs) and in HBMMSCs infected with rLV, rLV-miR675, respectively. β-actin as internal control.

FigureS2 Real-time RT-PCR analysis of mature miR675 in the human mesenchymal stem cells (HBMMSCs) and in HBMMSCs infected with rLV, rLV-miR675, respectively. U6 as internal control.
